# Epidemiology of moderate and severe traumatic brain injury in Cairo University Hospital in 2010

**DOI:** 10.1186/cc12258

**Published:** 2013-03-19

**Authors:** T Montaser, A Hassan

**Affiliations:** 1 Shobra General Hospital, Cairo, Egypt; 2 King Khaled University Hospital, Riyadh, Saudi Arabia

## Introduction

Traumatic brain injury (TBI) is a contributing factor to approximately one-third of all injury-related deaths in the USA annually. Updated statistical records for TBI in Egypt are lacking. The current research is aiming to estimate the prevalence of TBI in Egypt in order to develop a comprehensive TBI prevention program.

## Methods

A 1-year period (one calendar month every quarter of 2010) descriptive epidemiological study of moderate and severe TBI cases admitted to the emergency department, Cairo main university hospital. The data collection sheet included personal data (age, sex and residency), incident-related data (cause, nature and time of injury) and both clinical and radiological findings.

## Results

Table 1 shows the magnitude of the problem, highlighting the leading causes of TBI in Egypt in 2010. Male sex was predominantly affected, 79% of cases. Moderate and severe injuries account for 17.2% of all TBI presented cases.

## Conclusion

TBI is a serious public health problem in Egypt. Further data interpretation over wider periods of time should be conducted for better understanding of TBI prevalence is highly recommended to develop effective injury prevention programs.

**Table 1:** Causes of TBI in different age groups in Egypt

Age (years)	NO = 844/ 4 months	FFH	MVC	Abuse/ neglect	Violence/ assaults	Struck by/ against	Other causes/ not known
1 to 5	54 (6%)	20 (37%)	10 (19%)	8 (15%)	-	8 (15%)	8 (14%)
6 to 18	91 (11%)	28 (31%)	20 (22%)	5 (6%)	6 (5 %)	22 (25%)	10 (11%)
19 to 29	150 (18%)	49 (33%)	45 (30%)	-	14 (9%)	18 (12%)	24 (16%)
30 to 55	381 (45%)	114 (30%)	134 (35%)	-	19 (5%)	50 (13%)	64 (17%)
Above 55	168 (20%)	47 (28%)	41 (24%)	11 (7%)	5 (3%)	25 (15%)	39 (23%)

